# Effective emotion regulation and positive psychological capital as coping strategies to alleviate teacher burnout: a narrative review

**DOI:** 10.3389/fpsyg.2025.1639037

**Published:** 2025-09-10

**Authors:** Shicun Qiu, Jiacun Qiu, Jinwen Xu, Lin Wang

**Affiliations:** ^1^School of Foreign Languages, Sichuan University of Arts and Science, Dazhou, China; ^2^Business School, Guangdong Business and Technology University, Zhaoqing, China; ^3^International College, Chiang Mai Rajabhat University, Chiang Mai, Thailand; ^4^School of Foreign Languages, Southwest Medical University, Luzhou, China

**Keywords:** teacher burnout, coping strategies, effective emotion regulation, positive psychological capital, narrative review

## Abstract

In contemporary society, teacher burnout (TB) continues to be a prevalent issue within educational contexts, negatively impacting teacher job satisfaction and student outcomes. This study investigates the efficacy of positive coping strategies, including effective emotion regulation (EER) and positive psychological capital (PPC), in mitigating TB. Based on conceptual analysis and an extensive narrative review of current literature, the research explores the relationships between these coping strategies and TB. This narrative review highlights that the combined utilization of EER and PPC may present a more robust and holistic strategy for reducing TB than any of these elements can individually achieve. An integrative approach that simultaneously addresses these dimensions will harness their synergistic effects, fostering a more resilient, competent, and emotionally balanced teaching workforce. This review contributes to the broader understanding of TB by emphasizing the need for interventions and professional development programs that enhance EER and PPC among teachers. Implementing such coping strategies can significantly improve teacher job satisfaction and, subsequently, the educational environment. The review calls for further research to develop targeted interventions addressing TB, ultimately fostering a more supportive and effective teaching profession.

## 1 Introduction

The competence and effectiveness of teachers are universally acknowledged as essential for the success in the field of education (Pan et al., 2023). In contemporary society, the teaching profession holds substantial significance. Nonetheless, it is also associated with a high risk for teacher burnout (TB), attributed to the continuous emotional demands experienced by teachers (Derakhshan et al., 2020). This risk is exacerbated by numerous stressors, such as an increasing workload, extended working hours, physical exhaustion, emotional depletion, compassion fatigue, feelings of ineffectiveness, and cognitive fatigue, all contributing to TB (Fathi et al., 2021; Dai and Wang, 2023). Indeed, TB manifests as an incapacity to effectively manage anxiety related to work, a deterioration in interpersonal relationships, sustained feelings of exhaustion, and a decreasing motivation in the teaching career. Anyway, TB is a significant factor that adversely affects teachers' professional commitment and effectiveness (Pan et al., 2023).

Accordingly, teachers need to proficiently utilize positive psychological resources and effective methodologies to skillfully manage the emotions they often encounter in their professional settings, particularly those associated with TB. By developing positive coping strategies, teachers can mitigate TB, thereby enhancing their overall effectiveness (Myruski et al., 2018). These coping strategies are intrinsically linked to positive psychological resources such as effective emotion regulation (EER) and positive psychological capital (PPC). To begin with, access to EER is crucial for teachers to alleviate TB (Jiang et al., 2016), for EER enables teachers to manage negative emotions that hinder the attainment of educational goals (Myruski et al., 2018). As a vital role in reducing TB, PPC is also positively linked to effective coping strategies, which are in turn negatively correlated with TB. Those teachers with positive attitudes are more inclined to adopt constructive problem-solving approaches. Conversely, those teachers under stress often display negative emotions, which obstruct their engagement in practical work performance and aggravate TB consequently (Liu and Li, 2020).

However, the existing literature has shown that researchers have put much more effort into emphasizing the negative factors affecting TB, but the factors mitigating TB have been relatively neglected (Zhang et al., 2019; Ma and Liu, 2024). Less researchers have paid attention to the combined effects of positive psychological resources like PPC and EER exerted as coping strategies in lessening TB. To examine the proactive impact of each coping strategy as well as their combined effects, this narrative review aims to enrich our understanding of the importance and the way that each positive coping strategy exerts its influence and then integrates well together to alleviate TB.

## 2 Aim, search strategy, and selection process

Although there is not a universally accepted definition of burnout, it has been rigorously examined for more than five decades. The theoretical and empirical evidence delineated in this study illustrates that TB constitutes a multifaceted and pervasive challenge within educational contexts. This narrative review aims to synthesize this evidence, arguing that the combined utilization of EER and PPC presents a more robust and holistic strategy for reducing TB than any of these elements would individually achieve.

### 2.1 Search strategy

To ensure a comprehensive and unbiased retrieval of relevant literature, an extensive search of electronic databases including PsycINFO, PubMed, Scopus, and Web of Science was conducted in March 2025. The search strategy was built around core concepts: teacher burnout, coping strategy, emotion regulation, and psychological capital. The following search string, utilizing Boolean operators, was adapted for each database: (“burnout” OR “teacher burnout” OR “teacher turnover” OR “teacher stress”) AND (“coping strateg^*^” OR “emotion regulation” OR “psychological capital”). The inclusion criteria were limited to relevant journal articles and books, published in English between January 1974 and March 2025. We selected this date range to capture the seminal work of Freudenberger (1974) and all subsequent literature up to the time of our search. All study designs (e.g., theoretical, qualitative, quantitative, review articles) were considered eligible.

### 2.2 Study selection and screening

The initial database searches yielded a total of 2,347 records. After removing 658 duplicates, the titles and abstracts of 1,689 articles were screened against the inclusion and exclusion criteria. Exclusion criteria were: (a) studies not measuring or discussing burnout as a primary outcome, and (b) studies not available in full text. This screening process resulted in 213 articles for full-text assessment.

Then, two members of the research team independently evaluated these full-text articles for eligibility. Any disagreements were resolved through discussion until consensus was reached. The primary reasons for exclusion at this stage were a lack of focus on the positive psychological constructs of interest (EER and PPC) or a primary focus on student outcomes rather than teacher burnout. This process yielded a core set of 111 articles that directly informed the review.

Furthermore, to ensure a broad, inclusive perspective and to capture seminal works and diverse cultural contexts (e.g., the specific challenges faced by Chinese teachers, as discussed in Section 3.3), a secondary strategy of citation chasing (reviewing reference lists of key articles) and expert consultation was employed. This supplementary approach added 22 crucial publications, resulting in a final corpus of 133 publications that form the narrative synthesis of this review.

### 2.3 Narrative synthesis approach

As a narrative review, the goal was not to perform a meta-analysis but to provide a critical, thematic synthesis of the literature. The selected studies were analyzed to identify key themes, consensus findings, theoretical developments, and gaps in the literature, particularly regarding the interplay between EER, PPC, and TB. The structure of the review (conceptualization of TB, causes, effects, and coping strategies) emerged from this analytical process.

## 3 Conceptualizing teacher burnout

### 3.1 Burnout

As a very elusive concept, burnout is a syndrome that initially emerged in scholarly articles through descriptive and qualitative observations conducted by pioneering researchers in human services and healthcare during the mid-1970s (Chang, 2009). The psychological term *burnout* was first introduced by Freudenberger (1974) to describe definitive symptoms of emotional depletion as well as a decline in motivation and commitment. Subsequently, Maslach (1976) identified burnout as a pervasive phenomenon within caregiving and service professions, in which the fundamental interpersonal context for burnout involves the emotional, motivational, and value-based interactions between providers and recipients. Later, Maslach and Jackson (1981) conceptualized burnout as a syndrome characterized by high emotional exhaustion (EE) and depersonalization (DE), and reduced personal accomplishment (RPA). Furthermore, Pines and Aronson (1988) established a connection between burnout and emotional, mental, and physical exhaustion, which often results directly from prolonged exposure to emotionally demanding situations. In fact, burnout occurs more in interpersonally oriented occupations such as the teaching profession (Maslach and Leiter, 1999). To sum up, defined as a dysfunctional response to chronic emotional and interpersonal stressors at work, burnout is a psychological syndrome that causes a person to be under work-related stress and frustration for a long time (Wang and Guan, 2020; Derakhshan et al., 2022).

Although there is no standard definition, a clear consensus has been reached regarding the three core dimensions (EE, DE, and RPA) of the burnout experience. This consensus, along with subsequent research on the issue, has driven the multidimensional development of burnout theory (Maslach, 1998). To a great extent, the only measure encompassing these three core dimensions is the Maslach Burnout Inventory (MBI) which was developed by Maslach and Jackson (1981), and then the MBI-Human Services Survey (MBI-HSS) was designed for use by professionals in human services and healthcare. Subsequently, another version was developed for educators, known as the MBI-Educators Survey (MBI-ES). The labels for the three dimensions in both the HSS and ES forms reflect the focus on occupations where individuals extensively interact with others, such as clients, patients, and students (Maslach et al., 2001).

Generally speaking, EE is the hallmark of burnout and represents the most evident symptom of this multifaceted syndrome. Some scholars have even posited that the remaining two components are peripheral or superfluous, given EE's strong association with burnout (Shirom, 1989). Nevertheless, while EE is an essential criterion for burnout, it alone is insufficient, as it does not encapsulate the crucial aspects of individuals' relationships with their work environment (Maslach et al., 2001). In the realm of human services, the emotional demands of a job can and often do deplete a service provider's ability to engage with and respond to the needs of service recipients. DE involves a deliberate effort to distance oneself from service recipients by consciously ignoring the qualities that make them unique and engaging. This distancing serves as an immediate response to exhaustion, which explains the strong correlation between EE and DE in burnout studies (Maslach et al., 2001). Moreover, the association between RPA (or inefficacy) and the other two facets of burnout is notably intricate. In certain scenarios, it is indisputably attributable to depersonalization or a synthesis of both (Lee and Ashforth, 1996). A work environment characterized by persistent, overwhelming demands that contribute to EE or DE will undeniably undermine an individual's sense of efficacy. As a matter of fact, EE or DE directly hampers effectiveness. It is apparent that the lack of efficacy seems to arise more clearly from a lack of relevant resources, whereas EE and DE result from excessive workloads and social conflicts (Maslach et al., 2001). In a word, within the educational sector, DE emerges when teachers develop harmful and negative perceptions toward others. EE and RPA manifest when a teacher's productivity and professional competence significantly deteriorate (Maslach and Leiter, 2016).

### 3.2 Teacher burnout

Undoubtedly, TB is a widespread concern within the field of education. Intricately characterized by EE, DE, and RPA (Maslach et al., 2001; Chang, 2009), TB stems from persistent workplace stressors inherent in teaching, such as significant workloads, emotional demands, and challenging student behaviors (Maslach and Leiter, 2016; Li, 2023). Early scholars examined TB through the perspective of teacher stress (Smylie, 1999). In the early 1980s, researchers in the domain of teacher education conclusively identified the fundamental factors contributing to teacher stress and burnout based on demographic data, including sex, age, marital status, years of teaching experience, and educational level. By the late 1980s, studies began to explore work-related elements, such as teacher-student ratios, types of exceptional children taught, and overall workload. Throughout the 1990s, researchers utilized theoretical frameworks to investigate the interaction between TB and the work environment (Chang, 2009). In recent years, this interaction still attracts academic attention. For instance, empirical research by Gallego (2024) demonstrated that mathematics teachers exhibit significantly lower TB levels, particularly in EE and DE dimensions, when working within positive and supportive institutional environments.

The origins of TB, traceable to the late 1970s through the work of Maslach and her colleagues, unequivocally underscore the profession's unique demands (Maslach, 1976; Maslach et al., 2001). Nevertheless, a thorough analysis of these origins unveils the evolving educational landscape. Elements such as technological advancements, shifting societal expectations, and diverse student demographics are shaping contemporary challenges for teachers. Maslach's research on burnout has persisted as the predominant framework for examining burnout in recent decades, primarily because she formulated the key psychological constructs that have defined the conceptualization of burnout. Researchers have widely adopted the MBI scale, developed by Maslach and Jackson (1981), which assesses burnout across three dimensions (EE, DE, and RPA). In the teaching field, EE distinctly captures persistent feelings of emotional exhaustion and fatigue, underscoring the relentless struggle to meet the emotional demands of teaching. DE manifests as a disengaged attitude toward students and colleagues, inevitably resulting in a failure to recognize the unique needs and emotions of students. RPA represents a diminished sense of efficacy and accomplishment in the teaching role, which threatens professional self-esteem (Madigan et al., 2023).

TB represents a prolonged exposure to emotional and interpersonal stressors in the workplace, often coupled with inadequate recovery, leading previously dedicated teachers to disengage from their roles (Steinhardt et al., 2011). The literature consistently demonstrates that TB is associated with various psychosocial stressors, such as excessive workload (de Beer et al., 2015), interpersonal conflicts, and a deficiency in social support (Mede, 2009). This indicates that TB is a consequence of a toxic work environment and insufficient personal resources. To be specific, EE is directly related to heightened stress, anxiety, and depressive symptoms, whereas DE significantly impairs essential teacher-student relationships. RPA adversely affects job satisfaction and heightens turnover intentions, collectively undermining psychological resources (Pyhältö et al., 2021).

While the extant literature furnishes significant insights into the dimensions, origins, and repercussions of TB (Shen et al., 2015), an in-depth analysis underscores the necessity for a more nuanced comprehension and targeted interventions tailored to Chinese teachers. Elements such as cultural influences, linguistic barriers, and the unique challenges encountered by Chinese teachers remain insufficiently explored (Cheng et al., 2023). Moreover, it is essential to recognize that the issues confronted by Chinese teachers extend beyond the conventional stressors discussed in the existing literature. Cultural nuances, language barriers, and the distinct dynamics of language teaching undeniably add layers of complexity to these teachers' experiences. The present corpus of research does not adequately account for these distinctive factors, thereby limiting the applicability of current interventions and understanding of TB in this specific context (Liu and Du, 2024). It is crucial to investigate the interplay between positive coping strategies (such as EER and PPC) and TB within the Chinese educational context. This exploration will not only enrich the literature on TB but also offer tailored insights to inform interventions addressing the concern of TB.

### 3.3 Causes contributing to teacher burnout

It is a well-established fact that everyone encounters some degree of stress in their lives, particularly those in high-pressure professions (Skaalvik and Skaalvik, 2011). Accumulated stress over time ultimately leads to job burnout (Larrivee, 2012). Anxiety and frustration directly affect employee performance and their capacity to engage with colleagues (Sterrett et al., 2011). Specifically, teachers who are both emotionally and physically worn out often describe themselves as exhausted or drained when asked about their feelings (Goldhaber and Cowan, 2014; Ingersoll, 2012).

Teaching is a profession that is exceptionally vulnerable to burnout, especially in the form of EE (Akça and Yaman, 2010). Teachers are more prone to experiencing burnout compared to other white-collar professionals (Schaufeli and Enzmann, 1998). Various studies have effectively identified the sources of burnout, including interpersonal relationships (Jackson and Schuler, 1983), role conflict and ambiguity (Schwab and Iwanicki, 1982), and personal characteristics (Pretty et al., 1992). Researchers have pinpointed several factors contributing to job burnout, such as feelings of isolation, insufficient support from colleagues, overwhelming workload, lack of autonomy, disrespect from administrators, limited leadership opportunities, classroom management and discipline issues, and the emphasis on high-stakes testing and high achievement goals (Leiter and Maslach, 2005).

The role of gender in TB remains an understudied area in the literature, with scholars noting a significant research gap (Corrente et al., 2022). Existing findings present conflicting results: while some studies report no gender differences in TB levels (Kreuzfeld and Seibt, 2022), others document significant disparities. Some research suggests that male teachers exhibit higher TB levels than their female counterparts in countries like Turkey and Iran (Sak, 2018; Atashpanjeh et al., 2020), whereas other studies indicate that female teachers in countries such as Spain, Sweden, China, Ghana and Pakistan are more susceptible to TB (Sánchez-Pujalte et al., 2021; Stengård et al., 2022; Chen et al., 2023). Furthermore, female teachers report elevated trait anxiety, which may exacerbate TB (Levante et al., 2023). Although male and female teachers face comparable institutional stressors, societal expectations—particularly the dual burden of professional and traditional caregiving roles—may disproportionately influence TB trajectories among female teachers (Alghamdi and Sideridis, 2025).

TB itself is not a novel phenomenon; what is new is the increasing probability teachers experience burnout. On one hand, this condition is frequently associated with negative and cynical attitudes toward colleagues and work as a whole (Larrivee, 2012). On the other hand, insufficient resources and lack of administrative support undeniably exacerbate stress among educators. A number of teachers perceive themselves as unsupported and unfairly overloaded by impractical expectations (Lieberman and Friedrich, 2010).

From a pedagogical perspective, one of the most persistent challenges teachers encounter involves the ineffective management of demanding classroom situations. To a great extent, student misbehavior is undeniably one particular working condition significantly linked to TB. Teachers need to be adept at handling disciplinary issues to maintain an organized classroom environment and foster the learning and achievement of at-risk students. Those teachers who confront disruptive students often find themselves unable to teach effectively, which heightens their frustration (Aloe et al., 2014). RPA is associated with a lack of confidence and teaching efficacy, impeding their capacity to support students throughout the educational process (Maslach et al., 2001). Thus, teacher empowerment and self-efficacy have a direct effect on job satisfaction. Those teachers who feel empowered to exert control in their classrooms are more inclined to significantly influence student success (Sterrett et al., 2011). Similarly, insufficient collaboration with colleagues can also contribute to TB. When teachers perceive their professional relationships as dysfunctional, they tend to feel isolated, which elevates stress levels and ultimately leads to TB (Levine and Marcus, 2010; Skaalvik and Skaalvik, 2011). Moreover, personality traits also contribute to teacher isolation. According to Akkerman and Meijer (2011), individuals who are insecure, anxious, and ambivalent are more prone to burnout as they often isolate themselves and avoid interacting with their colleagues. Nayernia (2021) further indicated that teachers' language proficiency is negatively correlated with EE and DE.

Anyway, both personal and environmental determinants are fundamental causes of TB. Burnout, self-efficacy, job satisfaction, and contextual influences are all linked to the time pressures experienced by teachers and their relationships with parents. These elements are all significant predictors of EE and DE (Skaalvik and Skaalvik, 2010). Skaalvik and Skaalvik (2009) also definitively showed that all the subscales of burnout and teacher autonomy and support are negatively correlated. Teachers who do not feel burnout are more inclined to feel independent and more supportive of their students. Another study demonstrated that institutional supervision is a key factor in teachers' burnout, which showed that teachers who are monitored and supervised by institute authorities are more likely to feel burned out (Ghanizadeh and Ghonsooly, 2014). Conversely, there is a negative and significant correlation between EER and TB. Lauerman and König (2016) also asserted that the association between self-efficacy, pedagogical knowledge, and burnout is negative.

Taking Chinese university teachers as an example, they frequently encounter the difficulty of reconciling the competing obligations of teaching and research. If not managed properly, this can result in TB. Excessive demands in teaching or research can exhaust their emotional resources, compelling them to exert additional effort to balance these expectations. Furthermore, they often face unique job demands that can lead to TB due to limited resources, conflicting personality requirements, and reward systems (Xu, 2017).

### 3.4 Negative effects of teacher burnout

It is an incontrovertible reality that TB significantly detracts from both teacher job satisfaction and the educational milieu (Schaufeli and Bakker, 2004; Zhang et al., 2023). In the educational domain, EE contributes significantly to increased stress levels, anxiety, and manifestations of depression, while DE substantially disrupts fundamental pedagogical interactions between teachers and students. RPA negatively impacts teachers' professional fulfillment and amplifies their propensity to exit the teaching occupation, consequently compromising their overall psychological resources (Pyhältö et al., 2021).

TB is primarily a detrimental issue that adversely affects education quality as well as student performance. The manifestation of TB among teachers is significantly associated with increased incidences of emotional outbursts and diminished patience in classroom interactions, thereby adversely affecting student learning outcomes (Jacobson, 2016). With the intensification of TB symptoms, students experience academic and emotional distress due to their teachers' inefficacy and inconsistency (Shen et al., 2015; Jacobson, 2016). Additionally, TB can impact the job satisfaction of colleagues and the entire organization due to heightened teacher absenteeism, thereby imposing a considerable burden on an already strained workforce (Zeichner and Liston, 2013).

Research also demonstrates that occupational demands negatively impact employee health across various professions (Burgard and Lin, 2013). Similarly, this is the case for the teaching profession, as studies indicate that teachers are susceptible to many health consequences, which includes the development of mental health conditions like depression (Gray et al., 2017), as well as negative effects on their physical health such as an increased incidence of somatic symptoms and cardiovascular diseases (Scheuch et al., 2015). Worryingly, there is also evidence that teacher health is worsening, and those global public health emergencies like the COVID-19 pandemic has only exacerbated teachers' worsening health even further (Kim et al., 2022).

Teachers of different subjects may face varying levels of TB. McLean and Corbin's (2025) comparative study of American teachers across mathematics, English language arts, and science disciplines revealed distinct disciplinary variations in TB manifestations. Science teachers reporting higher DE and EE scores were associated with delivering shorter lessons overall, suggesting science education may represent a particularly burnout-sensitive pedagogical context. This phenomenon may stem from systemic resource deficiencies, wherein science teachers experiencing TB lacked critical support structures (including supportive colleagues, curricular materials, and prior training experiences) essential for maintaining both instructional duration and effective implementation of teacher-intensive grouping methodologies. As a whole, however, teachers exhibiting higher levels of TB tend to allocate less instructional time to whole-class and small-group formats while favoring independent instruction. This implies that TB influences pedagogical decision-making in a consistent manner across content areas, with TB levels shaping instructional choices.

## 4. Positive coping strategies to alleviate teacher burnout

This part aims to explore and explicate the complex relationships between the positive coping strategies (EER and PPC) and TB within educational settings. The primary goal is to identify effective coping mechanisms that can reduce the incidence of TB and make proper interventions. This research seeks to offer an in-depth understanding of how these positive coping strategies impact teachers' overall job satisfaction and contribute to the mitigation of TB.

One highly effective method to mitigate TB is through the application of positive coping strategies. Coping involves recognizing and articulating one's emotions while selecting appropriate methods to either amplify or diminish these feelings (Lazarus, 2000; Davis et al., 2008). The current body of literature indicates a significant shift in perspectives on coping. There is now a broad agreement that coping transcends a mere reaction to emotions, being intrinsically linked to the emotional process itself (Lazarus, 2000). In terms of coping strategies, teachers who predominantly employ avoidance techniques report elevated stress levels or burnout (Chang, 2009). On the contrary, positive coping serves as an efficacious mechanism for managing stress and emotions. This strategy is notably future-oriented, focusing on personal capacity in goal management, problem anticipation, and challenge-seeking, contrasting with risk management, which is reactive and seeks to compensate for loss or harm. Proactive individuals are not merely reactive; they do not interpret challenges as losses or harms but rather as neutral opportunities. Consequently, positive coping represents a proactive approach to handling stressors, integrating self-regulatory processes aimed at goal attainment.

As mentioned in the above parts, various individual and environmental factors fundamentally contribute to TB and its effect on teachers' job satisfaction and the educational environment is undeniable (Schaufeli and Bakker, 2004; Zhang et al., 2023). Hence, to effectively manage the adverse emotions associated with TB, teachers must skillfully utilize positive psychological resources. In fact, mastering positive coping strategies to combat TB is crucial for overcoming the difficulties faced by educators, ultimately contributing to their overall success (Myruski et al., 2018). The following two sections will examine the significance and necessity of employing positive coping strategies to mitigate TB, drawing on positive psychological resources such as EER and PPC.

### 4.1 Effective emotion regulation (EER)

Research distinguishes between EER strategies (e.g., cognitive reappraisal, acceptance) and emotion regulation (ER) difficulties (e.g., avoidance, expressive suppression). Experimental studies (Goldin et al., 2008; Aldao et al., 2010; Iuga and David, 2024) indicate that ER difficulties exacerbate psychological symptoms, whereas EER strategies reduce negative affect and burnout. A meta-analysis by Iuga and David (2024) further confirmed that EER negatively correlates with burnout, while ER difficulties positively associate with burnout. Thus, EER serves as a protective factor, whereas maladaptive regulation heightens vulnerability to burnout. Empirical evidence demonstrates a significant positive relationship between cognitive reappraisal and teaching satisfaction, along with an inverse association with EE (Tsouloupas et al., 2010). Supporting these findings, Chang and Taxer's (2020) investigation of trait-level ER revealed that teachers exhibiting higher reappraisal tendencies and lower levels of suppression reported minimal anger and EE symptoms.

Recently, numerous scholarly investigations have underscored the significance of EER (Ma and Liu, 2024; Sadoughi et al., 2024; Yang and Du, 2024). Specifically, Sadoughi et al. (2024) demonstrated the predictive role of EER in alleviating TB. To them, EER can mitigate TB as it involves reconstructing the interpretation of stressful situations to alter their negative emotions, whereas suppressing emotional regulation strategy may exacerbate TB due to its association with heightened psychological strain. In this way, it is imperative for teachers to regulate their emotions using effective coping strategies (Chang, 2009). In fact, those teachers who are adept at EER exhibit greater resilience against TB (Clipa and Boghean, 2015), and language teachers proficient in EER can navigate challenges and adversities more efficiently (Li, 2023). Consequently, those teachers capable of managing their emotions are less prone to experiencing EE, DE and RPA (Maslach and Leiter, 2016). Therefore, EER should exert influence over how teachers express emotions, manage stress, and interact with others, particularly within the context of TB (Lopes et al., 2004). Teachers who practice EER can anticipate benefits in multiple domains, including improved teaching quality, protection against burnout, and enhanced learning experiences for students (Chang, 2009). In a word, EER is vital for bolstering teachers' problem-solving capabilities and providing them with the cognitive and social resources necessary to mitigate TB (Ma and Liu, 2024). EER is theoretically conceptualized as the physiological, behavioral, and cognitive processes that enable individuals to modulate the experience and expression of both positive and negative emotions, which encompasses a diverse range of processes through which emotions are regulated (Gross and John, 2003). From a constructivist perspective, EER is viewed as an individual's capacity to control and respond aptly to emotional experiences (Bielak and Mystkowska-Wiertelak, 2022). Indeed, in the realm of teaching, EER holds undeniable significance in various aspects. By effectively regulating their emotions, teachers can achieve their objectives, including educational, cultural, pedagogical, and managerial goals (Chang, 2020).

In 2002, Gross introduced a framework for EER encompassing two methods: reappraisal and suppression. Reappraisal is a strategy that enables individuals to alter their perception of a situation to reduce its emotional impact. Conversely, suppression exerts minimal influence on unpleasant emotions as it can deplete cognitive resources, consequently impairing memory during the process of EER. In a comprehensive examination of classroom ER patterns (including reappraisal and suppression strategies), Chang's (2020) study of 561 U.S. Midwestern teachers (comprising 3% African American, 94.5% Caucasian-American, and approximately 2% Asian or Latino teachers) established that chronic expressive suppression significantly predicts all three TB dimensions: EE, DE, and RPA. Notably, such suppression showed the strongest correlation with DE, a particularly concerning finding as this maladaptive strategy not only compromises teacher job satisfaction but also precipitates professional detachment, potentially impairing teacher-student relationships and pedagogical engagement.

However, in the context of classroom management, Tsouloupas et al. (2010) once found no mediating role of cognitive reappraisal or expressive suppression in the relationship between perceived student misbehavior and EE among 610 K−12 teachers. The researchers hypothesized that the substantial proportion of veteran teachers (45% with over 11 years' experience) in their sample might have diminished the potential mediating effects of ER strategies. This finding aligns with Chang's (2013) study, which similarly failed to demonstrate the protective effects of cognitive reappraisal against TB when teachers encountered student misbehavior. Chang's work further revealed that frequent use of avoidance or suppression strategies actually increased vulnerability to TB. Taxer and Gross (2018) provide a theoretical explanation for these context-dependent findings, proposing that the efficacy of cognitive reappraisal may vary according to teachers' specific ER goals in classroom situations.

To a great extent, teachers often neglect or suppress their emotions due to the work and power dynamics within schools posing significant threats to their objectives. This impact is apparent in the manner in which teachers manifest intense emotional distress and anger (Liljestrom et al., 2007). Therefore, it is evident that self-regulatory knowledge is crucial for teachers' EER. As Chang (2009) asserts, ER difficulties such as expressive suppression, faking, or concealing true emotions contribute to increased burnout. Hence, teachers must thoroughly understand EER and coping strategies to manage the classroom environment effectively (Chang, 2009).

Anyway, the likelihood of experiencing burnout significantly increases for teachers lacking EER, particularly in the context of EE (Chang, 2009). Hence, a comprehensive understanding of their emotions is essential for teachers to regulate them effectively. As EER plays a crucial role in the teaching profession (Sutton and Wheatley, 2003), teachers need to realistically acknowledge the presence of unpleasant emotions in teaching and discard the notion that emotions are irrational or inappropriate in the classroom setting. It is imperative for teachers to accurately identify and label their emotional experiences. They should adopt a positive attitude toward emotions in the classroom and refrain from avoiding discussions about unpleasant emotions. In this way, reflecting on their feelings and the judgments they make is essential for teachers (Chang and Davis, 2009). Additionally, to manage their emotions effectively, teachers should employ proactive strategies to adjust their teaching goals based on a deeper understanding of student behavior and emotions. It is critical for teachers to reflect on the emotions and the judgments teachers are making about the events and then utilize positive strategies to regulate those negative emotions. Teachers must recognize that avoidance is the least effective method for addressing troubled person-environment issues and socializing with colleagues and school administration can significantly reduce TB and enhance success (Yang, 2022).

For instance, TB necessitates increased consideration within Chinese EFL teacher programs, as those teachers experiencing TB often show decreased motivation, reduced interest in teaching, increased exhaustion, and develop negative perceptions of their students (Ma and Liu, 2024). Bing et al. (2022) identified EER as a significant predictor of TB among EFL teachers, demonstrating that teachers with stronger regulatory abilities experience lower EE and DE while managing professional stressors more effectively. This aligns with Fathi et al.'s (2021) assertion that EER enhances EFL teachers' capacity to handle emotional demands, thereby mitigating EE and TB.

Thus, Chinese EFL teacher training programs can focus on enhancing practical skills and strategies for developing effective competencies among EFL teachers. Within this framework, EER is a crucial tool for teachers (Ma and Liu, 2024). Those EFL teachers who adeptly manage their emotions are better equipped to handle the emotional challenges associated with TB, such as frustration, disillusionment, and potential detachment from students. This emotional resilience can serve as a protective barrier against the development and escalation of TB. Furthermore, accounting for the significance of EER among teachers increases the likelihood of effectively reducing the occurrence of TB. Those teachers suffering from TB often exhibit reduced motivation, waning interest in teaching, heightened exhaustion, and develop negative attitudes toward their students (Ma and Liu, 2024). Therefore, it is crucial that teacher education programs within the Chinese EFL context emphasize the enhancement of practical skills and strategies to cultivate effective competencies among EFL teachers. Within this framework, EER serves as an indispensable tool for EFL teachers (Ma and Liu, 2024). Those EFL teachers adept at regulating their emotions are better positioned to manage the emotional dimensions which are often associated with TB, such as frustration, disillusionment, and even detachment from their students.

### 4.2 Positive psychological capital (PPC)

Positive psychology represents an established academic discipline that systematically examines human strengths and the cultivation of psychological potential. Building upon the theoretical foundations of positive psychology and positive organizational behavior theory, Luthans and Youssef (2004) pioneered the conceptualization of “positive psychological capital,” which underscores the pivotal function of positive psychological capacities in facilitating adaptive cognition and optimal functioning. Conceptualized as a higher-order construct comprising optimism, hope, resilience, and self-efficacy (Luthans et al., 2007), PPC serves as a protective factor against professional burnout (Freire et al., 2020). Although relatively stable, PPC is not fixed. Empirical evidence demonstrates its responsiveness to targeted interventions (Wang et al., 2025), positioning it as a dynamic, developable psychological resource rather than a static trait (Luthans and Youssef, 2004).

PPC represents a favorable psychological condition indicative of an individual's developmental progress. It serves as a personal internal work resource that replenishes one's energy, thereby aiding in behavior regulation and reducing the likelihood of job burnout (Nixon et al., 2011). Teachers possessing high levels of PPC are believed to effectively manage their adverse emotions, enabling them to stay motivated and achieve success (Derakhshan et al., 2022). To maintain dedication to their profession and withstand its challenges, teachers need to operate within a psychologically supportive environment.

PPC functions as a protective barrier against burnout, comprising a collection of positive psychological resources, including self-efficacy, hope, optimism, and resilience, which enable individuals to confront challenges and adversities successfully (Gong et al., 2019). Within educational contexts, enhanced PPC equips teachers with the skills and self-assurance required to handle classroom complexities, maintain an optimistic outlook despite difficulties, foster hope among their students, and resiliently adapt to professional demands, which diminishes their vulnerability to TB (Zhang et al., 2019; Liu and Du, 2024). Higher levels of PPC endow teachers with critical resources essential for effectively navigating the intrinsic stressors of the teaching profession (Kaya and Altinkurt, 2018). PPC empowers teachers to manage job demands proficiently, thereby alleviating TB (Luthans et al., 2007). Empirical evidence consistently corroborates this assertion, demonstrating a link between PPC and reduced TB levels (Demir, 2018; Zhang et al., 2019).

Teachers with higher self-efficacy demonstrate greater competence in classroom organization, student management, and instructional monitoring, which correlates with increased job satisfaction and reduced TB (Bing et al., 2022). This aligns with the broader framework of psychological capital, wherein hope, self-efficacy, resilience, and optimism collectively represent fundamental psychological resources that facilitate goal achievement (Luthans et al., 2007). These four components share three core characteristics: an internalized sense of control, deliberate intentionality, and active goal pursuit, they also collectively enable “*positive appraisal of circumstances and probability for success based on motivated effort and perseverance*” (Luthans et al., 2007, p. 550). The synergistic operation of these components creates a dynamic system: optimism fosters positive outcome expectations, self-efficacy drives goal selection and commitment, hope generates alternative pathways when facing obstacles, and resilience enables recovery from setbacks (Luthans and Youssef-Morgan, 2017). This integrated system maintains motivation and perceived control throughout goal pursuit processes.

Despite their interconnectedness, each component exhibits distinct characteristics. Hope, self-efficacy, and the future-oriented aspect of optimism are predominantly proactive traits, while resilience and the explanatory style dimension of optimism represent reactive responses to encountered situations (Luthans et al., 2007). Furthermore, pathway generation is unique to hope, distinguishing it from other components. The resources also differ in their orientation: hope and self-efficacy focus primarily on internal cognitive processes, whereas optimism and resilience incorporate external attributions and social resources, respectively (Luthans and Youssef-Morgan, 2017).

Having employed Structural Equation Modeling to examine the relationships among psychological capital and teacher burnout based on a sample of 387 Chinese English as a Foreign Language (EFL) teachers in primary and secondary schools, Liu and Du (2024) revealed a significant direct negative association between PPC and TB. This discovery reinforces the theoretical premises suggesting that elevated PPC levels provide individuals with crucial psychological resources necessary for effectively navigating and managing stressors, thereby mitigating burnout (Luthans et al., 2007; Demir, 2018; Zhang et al., 2019). The persistent support for this negative correlation between PPC and TB seems to be evident, as numerous other studies have consistently demonstrated that higher PPC is strongly correlated with reduced burnout levels among teachers (Demir, 2018; Kaya and Altinkurt, 2018; Zhang et al., 2019).

The theoretical foundation of PPC is deeply rooted in the principles of positive psychology. It posits that teachers possessing elevated levels of these beneficial psychological resources are exceptionally well-prepared to address challenges, adapt to stressors, and sustain an optimistic outlook (Luthans et al., 2007; Newman et al., 2014). This collection of positive traits functions as a protective mechanism, guarding educators against the harmful effects of burnout encountered due to the inherent rigors and demands of their profession (Kaya and Altinkurt, 2018; Zhang et al., 2019; Liu and Du, 2024). Theoretical frameworks unequivocally illustrate that the protective function of PPC depends on the presence of augmented psychological resources. Those teachers with elevated resilience are inclined to establish ambitious objectives, exhibit greater perseverance when confronted with challenges, and display heightened resistance to stressors. This collective strength serves as a safeguard against TB, enabling teachers to more adeptly navigate the complexities of their profession (Skaalvik and Skaalvik, 2007).

Therefore, it is essential to incorporate professional development programs aimed at augmenting teachers' PPC. Such programs should place a strong emphasis on the mental satisfaction of the teaching workforce. They ought to provide stress management strategies and interventions designed to bolster resilience and hope. Educational policies must tackle the incidence of TB. This can be accomplished by introducing measures to manage workloads, establishing robust support systems for teachers experiencing burnout, and offering incentives to enhance PPC among teachers (Liu and Du, 2024).

At last, gender and school type factors are worth considering. The relationship between gender and TB remains inconclusive. While Li (2016) reported greater EE among female EFL teachers in China, Sun and Dapat's (2024) study of 297 Chinese EFL teachers revealed no significant gender differences in hope, optimism, or overall PPC levels, though male teachers demonstrated significantly higher self-efficacy than their female counterparts, suggesting potential gender-based variations in this specific dimension. Then, taking school type-based variations into consideration, Sun and Dapat (2024) identified that private school teachers exhibiting higher levels of EE, DE, and RPA compared to public school educators. Private school teachers also reported lower hope and resilience. However, both sectors demonstrated comparable levels of self-efficacy and optimism, contrasting with previous findings that public sector employees typically show higher self-efficacy while private sector workers display greater optimism (Dirzyte and Patapas, 2022; Ji et al., 2022).

## 5 Discussion

The theoretical and empirical evidence delineated in this paper illustrates that TB constitutes a multifaceted and pervasive challenge within educational contexts. Since Freudenberger introduced the term in 1974, *burnout* has been rigorously examined for more than five decades. Although there is not a universally accepted definition of burnout, the three core dimensions (EE, DE, and RPA) conceptualized by Maslach and Jackson (1981) has become widespread consensus among researchers. As a pervasive concern in education, TB is also intricately characterized by these three core dimensions of EE, DE and RPA (Maslach et al., 2001; Chang, 2009). Based on an extensive review of the existing literature, we find that researchers have put much more attention to negative factors influencing TB than the proactive elements that help mitigate TB (Zhang et al., 2019; Ma and Liu, 2024). Hence, following the conceptualization of TB, as well as an examination of its causes and effects, this narrative review has emphasized the importance of positive coping strategies such as EER and PPC in alleviating TB. In fact, these coping strategies have been demonstrated a well-established association with TB. For instance, EER plays a crucial role by aiding teachers in managing their emotional responses to stressors, thus reducing TB (Gross, 2002; Clipa and Boghean, 2015; Ma and Liu, 2024). PPC, encompassing self-efficacy, hope, resilience, and optimism, has also been found to significantly reduce levels of TB (Luthans et al., 2007; Gong et al., 2019; Liu and Du, 2024).

Regarding the differences in TB across different educational stages, K−12 (or pre-university) teachers report higher TB levels than college teachers. Research indicates that approximately one-third of K−12 teachers quit within their first 3 years due to stress-induced burnout (McCarthy et al., 2014). While prior studies have identified a negative association between resource availability and TB among K−12 teachers (e.g., McCarthy et al., 2014), this relationship does not appear to hold for college teachers. This discrepancy may stem from unmeasured variables (such as workload demands) that uniquely influence stress experiences in higher education settings. Consequently, although college teachers report TB, their coping mechanisms appear to diverge from those documented in earlier research (Janczak, 2017).

During the COVID-19 pandemic, Brazilian elementary and secondary teachers exhibited higher TB rates than their higher education counterparts, whereas early childhood teachers reported the lowest levels (Ramos et al., 2023). Secondary teachers in the Philippines were classified as high TB due to the challenges of modular and distance learning (Malquisto et al., 2023). In fact, TB impairs teachers' ability to establish meaningful student-teacher relationships, compromising instructional quality (Guidetti et al., 2018). This issue is especially acute in secondary education, where job pressures intensify burnout symptoms, including emotional exhaustion (EE), depersonalization (DE), and reduced personal accomplishment (RPA) (García-Carmona et al., 2018).

The K−12 teaching environment is frequently characterized by isolation, a narrow focus on daily tasks, and limited opportunities for reflective practice. This professional isolation can lead to frustration, boredom, and emotional depletion as teachers contend with unaddressed anxieties (Fullan, 2001). Additionally, managing student misbehavior often leaves K−12 teachers intellectually and emotionally drained (Chang and Davis, 2009). Hence, high turnover rates linked to TB remain a pervasive issue in K−12 education worldwide (Liu and Onwuegbuzie, 2012). Fostering collaborative workplace cultures is critical to mitigating these challenges. For instance, effective teacher collaboration correlates with improved student outcomes in both mathematics and reading (Ronfeldt et al., 2015). Anyway, primary and secondary educators should prioritize institutional narratives that emphasize knowledge-sharing and technical collaboration among educators (Califf and Brooks, 2020).

On the whole, a significant fact is that TB is related with teacher isolation, which is a feeling of separation or actual separation while working with colleagues (Marshall, 2013), and collaborative environments help eliminate the feel of stress associated with working in isolation. In this way, teachers who benefited from a collaborative environment were more inclined to remain in the field of education (Darling-Hammond et al., 2012), as they feel valued and happier in a collaborative atmosphere so as to treat each other as well as their students in a more respectful manner (Jacobson, 2016). EER and PPC are both helpful to create this kind of collaborative environment.

Furthermore, the combined utilization of EER and PPC may present a more robust and holistic strategy for reducing TB than any of these elements would individually achieve. An integrative approach that simultaneously addresses these dimensions will harness their synergistic effects, fostering a more resilient, competent, and emotionally balanced teaching workforce. Research has demonstrated that EER serves as a positive predictor of PPC. In their investigation of 328 Chinese undergraduate and postgraduate students across diverse geographical regions during the COVID-19 pandemic, Tang and He (2022) found that the maintenance of EER may prevent the depletion of psychological resources and facilitate more efficient recovery from TB. In a study employing Structural Equation Modeling, Bing et al. (2022) examined the interrelationships among teacher self-efficacy, ER, and TB among 174 EFL teachers from various Chinese provinces. The analysis revealed that teacher self-efficacy accounted for 20% of the variance in TB levels, while ER explained an additional 11.2% of the variance. These results indicate that although both constructs significantly contribute to TB prediction, teacher self-efficacy emerges as a comparatively stronger determinant than ER capacity. Further supporting these findings, Ma's (2023) mediation analysis of 486 Chinese primary and secondary school teachers highlighted the critical role of EER and PPC in promoting their teaching engagement. The study underscores the potential benefits of targeted interventions aimed at enhancing these psychological resources among educational professionals.

Yet, implementing such a holistic approach requires a coordinated effort from educational leaders, policymakers, and stakeholders. Schools must prioritize mental health, providing comprehensive support systems, professional development programs, and a culture that values and acknowledges the emotional labor inherent in teaching. This will enhance teacher retention and satisfaction, thereby improving the overall quality of education for students.

In summary, TB remains a significant challenge, but the implementation of positive coping strategies such as EER and PPC shows potential in mitigating its adverse effects. This study has highlighted the critical significance of these elements in enhancing teachers' job satisfaction and promoting a sustainable teaching career. Future research may continue to explore novel intervention strategies and the practical applications of these strategies, ensuring that teachers are equipped with the necessary tools to thrive in their demanding yet rewarding profession.

## References

[B1] Akça F. Yaman B. (2010). The effects of internal-external locus of control variables on burnout levels of teachers. Procedia Soc. Behav. Sci. 2, 3976–3980. 10.1016/j.sbspro.2010.03.626

[B2] Akkerman S. F. Meijer P. C. (2011). A dialogical approach to conceptualizing teacher identity. Teach. Teach. Educ., 27, 308–319. 10.1016/j.tate.2010.08.01335237217

[B3] Aldao A. Nolen-Hoeksema S. Schweizer S. (2010). Emotion-regulation strategies across psychopathology: a meta-analytic review. Clin. Psychol. Rev., 30, 217–237. 10.1016/j.cpr.2009.11.004 20015584

[B4] Alghamdi M. H. Sideridis G. (2025). Identifying subgroups of teacher burnout in elementary and secondary schools: the effects of teacher experience, age and gender. Front. Psychol. 15:1406562. 10.3389/fpsyg.2024.1406562 39872728 PMC11770450

[B5] Aloe A. M. Amo L. C. Shanahan M. E. (2014). Classroom management self-efficacy and burnout: a multivariate meta-analysis. Educ. Psychol. Rev. 26, 101–126. 10.1007/s10648-013-9244-0

[B6] Atashpanjeh A. Shekarzehi S. Zare-Behtash E. Ranjbaran F. (2020). Burnout and job dissatisfaction as negative psychological barriers in school settings: a mixed-methods investigation of Iranian teachers. J. Educ. Health Promot. 9:334. 10.4103/jehp.jehp_583_20 33575370 PMC7871957

[B7] Bielak J. Mystkowska-Wiertelak A. (2022). Language teachers' interpersonal learner-directed emotion- regulation strategies. Lang. Teach. Res. 26, 1082–1105. 10.1177/136216882091235227409075

[B8] Bing H. Sadjadi B. Afzali M. Fathi J. (2022). Self-efficacy and emotion regulation as predictors of teacher burnout among English as a foreign language teachers: a structural equation modeling approach. Front. Psychol. 13:900417. 10.3389/fpsyg.2022.900417 35664188 PMC9160989

[B9] Burgard S. A. Lin K. Y. (2013). Bad jobs, bad health? How work and working conditions contribute to health disparities. Am. Behav. Sci. 57, 1105–1127. 10.1177/0002764213487347 24187340 PMC3813007

[B10] Califf C. B. Brooks S. (2020). An empirical study of techno-stressors, literacy facilitation, burnout, and turnover intention as experienced by K−12 teachers. Comput. Educ. 157:103971. 10.1016/j.compedu.2020.103971

[B11] Chang M-. L. (2009). An appraisal perspective of teacher burnout: examining the emotional work of teachers. Educ. Psychol. Rev. 21, 193–218. 10.1007/s10648-009-9106-y

[B12] Chang M-. L. (2013). Toward a theoretical model to understand teacher emotions and teacher burnout in the context of student misbehavior: appraisal, regulation and coping, Motiv. Emot. 37, 799–817. 10.1007/s11031-012-9335-0

[B13] Chang M-. L. (2020). Emotion display rules, emotion regulation, and teacher burnout. Front. Educ. 5:90. 10.3389/feduc.2020.00090

[B14] Chang M-. L. Davis H. A. (2009). “Understanding the role of teacher appraisals in shaping the dynamics of their relationships with students: Deconstructing teachers' judgments of disruptive behavior/students”, in Advances in Teacher Emotions Research, eds. Schutz P. A. Zembylas M. (New York: Springer).

[B15] Chang M-. L. Taxer J. (2020). Teacher emotion regulation strategies in response to classroom misbehavior. Teach. Teach. 27, 353–369. 10.1080/13540602.2020.1740198

[B16] Chen S. Ntim S. Y. Zhao Y. Qin J. (2023). Characteristics and influencing factors of early childhood teachers' work stress and burnout: a comparative study between China, Ghana, and Pakistan. Front. Psychol. 14:1115866. 10.3389/fpsyg.2023.1115866 36968706 PMC10038079

[B17] Cheng H. Fan Y. Lau H. (2023). An integrative review on job burnout among teachers in China: implications for human resource management. Int. J. Hum. Resour. Manag. 34, 529–561. 10.1080/09585192.2022.2078991

[B18] Clipa O. Boghean A. (2015). Stress factors and solutions for the phenomenon of burnout of preschool teachers. Proc. Soc. Behav. Sci. 180, 907–915. 10.1016/j.sbspro.2015.02.241

[B19] Corrente M. Ferguson K. Bourgeault I. L. (2022). Mental health experiences of teachers: a scoping review. J. Teach. Learn. 16, 23–43. 10.22329/jtl.v16i1.6856

[B20] Dai K. Wang Y. (2023). Investigating the interplay of Chinese EFL teachers' proactive personality, flow, and work engagement. J. Multilingual Multicult. Dev. 16, 209–223. 10.1080/01434632.2023.2174128

[B21] Darling-Hammond L. Amrein-Beardsley A. Haertel E. Rothstein J. (2012). Evaluating teacher evaluation. Phi Delta Kappan 93, 8–15. 10.1177/003172171209300603

[B22] Davis H. A. DiStefano C. Schutz P. A. (2008). Identifying patterns of appraising tests in first-year college students: implications for anxiety and emotion regulation during test taking. J. Educ. Psychol. 100, 942–960. 10.1037/a0013096

[B23] de Beer L. T. Pienaar J. Rothmann S. (2015). Work overload, burnout, and psychological ill-health symptoms: a three-wave mediation model of the employee health impairment process. Anxiety Stress Coping 29, 387–399. 10.1080/10615806.2015.1061123 26079200

[B24] Demir S. (2018). The relationship between psychological capital and stress, anxiety, burnout, job satisfaction, and job involvement. Eurasian J. Educ. Res. 75, 137–153. 10.14689/ejer.2018.75.8

[B25] Derakhshan A. Coombe C. Arabmofrad A. Taghizadeh M. (2020). Investigating the effects of English language teachers' professional identity and autonomy in their success. Issues Lang. Teach. 9, 1–28. 10.22054/ilt.2020.52263.496

[B26] Derakhshan A. Eslami Z. R. Curle S. Zhaleh K. (2022). Exploring the validity of immediacy and burnout scales in an EFL context: the predictive role of teacher-student interpersonal variables in university students' experience of academic burnout. Stud. Second Lang. Learn. Teach. 12, 87–115. 10.14746/ssllt.2022.12.1.5

[B27] Dirzyte A. Patapas A. (2022). Positive organizational practices, life satisfaction, and psychological capital in the public and private sectors. Sustainability 14:488. 10.3390/su14010488

[B28] Fathi J. Greenier V. Derakhshan A. (2021). Self-efficacy, reflection, and burnout among Iranian EFL teachers: the mediating role of emotion regulation. Iran. J. Lang. Teach. Res. 9, 13–37. 10.30466/ijltr.2021.12104335360565

[B29] Freire C. Ferradás M. D. M. García-Bértoa A. Núñez J. C. Rodríguez S. Piñeiro I. (2020). Psychological capital and burnout in teachers: the mediating role of flourishing. Int. J. Environ. Res. Public Health 17:8403. 10.3390/ijerph17228403 33202826 PMC7697347

[B30] Freudenberger H. J. (1974). Staff burn-out. J. Soc. Issues 30, 159–165. 10.1111/j.1540-4560.1974.tb00706.x

[B31] Fullan M. (2001). The New Meaning of Educational Change. New York: Teachers College.

[B32] Gallego M. K. F. (2024). School climate and burnout among mathematics teachers as basis for teacher empowerment action plan: a convergent parallel design. Am. J. Multidis. Res. Innov. 3, 13–27. 10.54536/ajmri.v3i4.2849

[B33] García-Carmona M. Marín M. D. Aguayo R. (2018). Burnout syndrome in secondary school teachers: a systematic review and meta-analysis. Soc. Psychol. Educ. 22, 189–208. 10.1007/s11218-018-9471-934573192

[B34] Ghanizadeh A. Ghonsooly B. (2014). A tripartite model of EFL teacher attributions, burnout, and self-regulation: toward the prospects of effective teaching. Educ. Res. Policy Pract. 13, 145–166. 10.1007/s10671-013-9155-3

[B35] Goldhaber D. Cowan J. (2014). Excavating the teacher pipeline: teacher preparation programs and teacher attrition. J. Teach. Educ. 65, 449–462. 10.1177/0022487114542516

[B36] Goldin P. R. McRae K. Ramel W. Gross J. J. (2008). The neural bases of emotion regulation: reappraisal and suppression of negative emotion. Biol. Psychiatry 63, 577–586. 10.1016/j.biopsych.2007.05.031 17888411 PMC2483789

[B37] Gong Z. Chen Y. Wang Y. (2019). The influence of emotional intelligence on job burnout and job performance: mediating effect of psychological capital. Front. Psychol. 10:2707. 10.3389/fpsyg.2019.02707 31920783 PMC6916327

[B38] Gray C. Wilcox G. Nordstokke D. (2017). Teacher mental health, school climate, inclusive education and student learning: a review. Can. Psychol. 58, 203–210. 10.1037/cap0000117

[B39] Gross J. J. (2002). Emotion regulation: affective, cognitive, and social consequences. Psychophysiology 39, 281–291. 10.1017/S0048577201393198 12212647

[B40] Gross J. J. John O. P. (2003). Individual differences in two emotion regulation processes: implications for affect, relationships, and well-being. J. Pers. Soc. Psychol. 85, 348–362. 10.1037/0022-3514.85.2.348 12916575

[B41] Guidetti G. Viotti S. Hindrichs I. Camacho-Avila A. Girardo C. Simon D. C.. (2018). Quality of life in the school context: the relationship between teachers' and students' wellbeing. Mediterr. J. Soc. Sci. 9, 117–127. 10.2478/mjss-2018-0143

[B42] Ingersoll R. M. (2012). Beginning teacher induction: what the data tell us. Phi Delta Kappan 93, 47–51. 10.1177/003172171209300811

[B43] Iuga I. A. David O. A. (2024). Emotion regulation and academic burnout among youth: a quantitative meta-analysis. Educ. Psychol. Rev. 36:106. 10.1007/s10648-024-09930-w

[B44] Jackson S. E. Schuler R. S. (1983). Preventing Employee Burnout. Personnel 60, 58–68. 10261205

[B45] Jacobson D. A. (2016). Causes and Effects of Teacher Burnout (Doctoral dissertation). Minneapolis, MN: Walden University.

[B46] Janczak R. R. (2017). Coping Strategies of College Faculty Experiencing Stress and Burnout at Work (Doctoral dissertation). Menomonie, WI: University of Wisconsin-Stout.

[B47] Ji J. Zhou L. Wu Y. Zhang M. (2022). Hope and life satisfaction among Chinese shadow education tutors: the mediating roles of positive coping and perceived social support. Front. Psychol. 13:929045. 10.3389/fpsyg.2022.929045 36081721 PMC9447439

[B48] Jiang J. Vauras M. Volet S. Wang Y. (2016). Teachers' emotions and emotion regulation strategies: self- and students' perceptions. Teach. Teach. Educ. 54, 22–31. 10.1016/j.tate.2015.11.008

[B49] Kaya Ç. Altinkurt Y. (2018). Role of psychological and structural empowerment in the relationship between teachers' psychological capital and their levels of burnout. Egitim ve Bilim 43:6961. 10.15390/EB.2018.6961

[B50] Kim L. E. Oxley L. Asbury K. (2022). “My brain feels like a browser with 100 tabs open”: a longitudinal study of teachers' mental health and well-being during the COVID-19 pandemic. Br. J. Educ. Psychol. 92, 299–318. 10.1111/bjep.12450 34337737 PMC8420299

[B51] Kreuzfeld S. Seibt R. (2022). Gender-specific aspects of teachers regarding working behavior and early retirement. Front. Psychol. 13:829333. 10.3389/fpsyg.2022.829333 35242087 PMC8887565

[B52] Larrivee B. (2012). Cultivating Teacher Renewal: Guarding Against Stress and Burnout. Landham, MD: Rowman and Littlefield Education Publication.

[B53] Lauerman F. König J. (2016). Teachers' professional competence and well-being: understanding the links between general pedagogical knowledge, self-efficacy and burnout. Learn. Instruct. 45, 9–19. 10.1016/j.learninstruc.2016.06.006

[B54] Lazarus R. S. (2000). Toward better research on stress and coping. Am. Psychol., 55, 665–673. 10.1037/0003-066X.55.6.665 10892209

[B55] Lee R. T. Ashforth B. E. (1996). A meta-analytic examination of the correlates of the three dimensions of job burnout. J. Appl. Psychol. 81, 123–133. 10.1037/0021-9010.81.2.123 8603909

[B56] Leiter M. P. Maslach C. (2005). Banishing Burnout: Six Strategies for Improving Your Relationship with Work. San Francisco, CA: Jossey-Bass.

[B57] Levante A. Petrocchi S. Bianco F. Castelli I. Lecciso F. (2023). Teachers during the COVID-19 era: the mediation role played by mentalizing ability on the relationship between depressive symptoms, anxious trait, and job burnout. Int. J. Environ. Res. Public Health 20:859. 10.3390/ijerph20010859 36613181 PMC9820251

[B58] Levine T. H. Marcus A. S. (2010). How the structure and focus of teachers' collaborative activities facilitate and constrain teacher learning. Teach. Teach. Educ. 26, 389–398. 10.1016/j.tate.2009.03.001

[B59] Li J. (2016). A survey on burnout and its influencing factors in the career development of college English teachers. Educ. Vocat. 4, 62–64. 10.13615/j.cnki.1004-3985.2016.04.01938566801

[B60] Li S. (2023). The effect of teacher self-efficacy, teacher resilience, and emotion regulation on teacher burnout: a mediation model. Front. Psychol. 14:1185079. 10.3389/fpsyg.2023.1185079 37691805 PMC10483148

[B61] Lieberman A. Friedrich L. D. (2010). How Teachers Become Leaders: Learning from Practice and Research. New York, NY: Teachers College Press.

[B62] Liljestrom A. Roulston K. deMarrais K. (2007). “‘There is no place for feeling like this in the workplace': Women teachers' anger in school settings,” in Emotions in Education, eds. Schutz P. A. Pekrun R. (San Diego: Elsevier).

[B63] Liu D. Du R. (2024). Psychological capital, mindfulness, and teacher burnout: insights from Chinese EFL educators through structural equation modeling. Front. Psychol. 15:1351912. 10.3389/fpsyg.2024.1351912 38550638 PMC10973147

[B64] Liu H. Li Z. (2020). “High school teacher retention” in Exploring Teacher Recruitment and Retention. eds. Ovenden-Hope T. assy R. P (London: Routledge), 176–184.

[B65] Liu S. Onwuegbuzie A. J. (2012). Chinese teachers' work stress and their turnover intention. Int. J. Educ. Res. 53, 160–170. 10.1016/j.ijer.2012.03.006

[B66] Lopes P. N. Brackett M. A. Nezlek J. B. Schütz A. Sellin I. Salovey P.. (2004). Emotional intelligence and social interaction. Pers. Soc. Psychol. Bull. 30, 1018–1034. 10.1177/0146167204264762 15257786

[B67] Luthans F. Avolio B. J. Avey J. B. Norman S. M. (2007). Positive psychological capital: measurement and relationship with performance and satisfaction. Pers. Psychol. 60, 541–572. 10.1111/j.1744-6570.2007.00083.x

[B68] Luthans F. Youssef C. M. (2004). Human, social, and now positive psychological capital management: investing in people for competitive advantage. Organ. Dyn. 33, 143–160. 10.1016/j.orgdyn.2004.01.003

[B69] Luthans F. Youssef-Morgan C. M. (2017). Psychological capital: an evidence-based positive approach. Ann. Rev. Organ. Psychol. Organ. Behav. 4, 339–366. 10.1146/annurev-orgpsych-032516-113324

[B70] Ma Y. (2023). Boosting teacher work engagement: the mediating role of psychological capital through emotion regulation. Front. Psychol. 14:1240943. 10.3389/fpsyg.2023.1240943 37720646 PMC10501726

[B71] Ma Y. Liu Z. (2024). Emotion regulation and well-being as factors contributing to lessening burnout among Chinese EFL teachers. Acta Psychologica 245:104219. 10.1016/j.actpsy.2024.104219 38492353

[B72] Madigan D. J. Kim L. E. Glandorf H. L. Kavanagh O. (2023). Teacher burnout and physical health: a systematic review. Int. J. Educ. Res. 119:102173. 10.1016/j.ijer.2023.102173

[B73] Malquisto I. R. Casinillo L. F. Salabao A. A. (2023). Burnout among secondary teachers amid the new normal: case in Ormoc City, Philippines. J. Res. Polic. Pract. Teach. Teach. Educ. 13, 28–39. 10.37134/jrpptte.vol13.12.2.2023

[B74] Marshall K. (2013). Rethinking Teacher Supervision and Evaluation: How to Work Smart, Build Collaboration, and Close the Achievement Gap (Second Edition). San Francisco, CA: Jossey-Bass.

[B75] Maslach C. (1976). BURNED-OUT. Hum. Behav. 5, 16–22.

[B76] Maslach C. (1998). “A multidimensional theory of burnout”, in Theories of Organizational Stress, ed. Cooper C. L. (Oxford, UK: Oxford Univ. Press).

[B77] Maslach C. Jackson S. E. (1981). The measurement of experienced burnout. J. Occup. Behav. 2, 99–113. 10.1002/job.4030020205

[B78] Maslach C. Leiter M. P. (1999). “Teacher burnout: A research agenda”, in Understanding and Preventing Teacher Burnout: A Sourcebook of International Research and Practice, eds. Vandenberghe R. Huberman A. M. (Cambridge, UK: Cambridge University Press).

[B79] Maslach C. Leiter M. P. (2016). Understanding the burnout experience: recent research and its implications for psychiatry. World Psychiatry 15, 103–111. 10.1002/wps.20311 27265691 PMC4911781

[B80] Maslach C. Schaufeli W. B. Leiter M. P. (2001). Job burnout. Annu. Rev. Psychol. 52, 397–422. 10.1146/annurev.psych.52.1.397 11148311

[B81] McCarthy C. J. Lambert R. G. Reiser J. (2014). Vocational concerns of elementary teachers: stress, job satisfaction, and occupational commitment. J. Employ. Counsel. 51, 59–74. 10.1002/j.2161-1920.2014.00042.x

[B82] McLean L. Corbin C. (2025). Associations among elementary teachers' burnout and their lesson length and classroom grouping in mathematics, science, and English language arts. Teach. Teach. Educ. 156:104937. 10.1016/j.tate.2025.104937

[B83] Mede E. (2009). An analysis of relations among personal variables, perceived self-efficacy and social support on burnout among Turkish EFL Teachers. Inonu Univ. J. Facul. Educ. 10, 39–52.

[B84] Myruski S. Denefrio S. Dennis-Tiwary T. (2018). “Stress and emotion regulation: The dynamic fit model”, in The Oxford Handbook of Stress and Mental Health, eds. Harkness K. L. Hayden E. P. (Oxford, UK: Oxford Univ. Press).

[B85] Nayernia A. (2021). “Language teacher burnout”, in Research Questions in Language Education and Applied Linguistics, eds. Mohebbi H. Coombe C. (New York: Springer).

[B86] Newman A. Ucbasaran D. Zhu F. E. I. Hirst G. (2014). Psychological capital: a review and synthesis. J. Organ. Behav. 35, S120–S138. 10.1002/job.1916

[B87] Nixon A. E. Yang L. Spector P. E. Zhang X. (2011). Emotional labor in China: do perceived organizational support and gender moderate the process? Stress and Health 27, 289–305. 10.1002/smi.1359

[B88] Pan Z. Wang Y. Derakhshan A. (2023). Unpacking Chinese EFL students' academic engagement and psychological well-being: the roles of language teachers' affective scaffolding. J. Psycholinguist. Res. 52, 1799–1819. 10.1007/s10936-023-09974-z 37249799

[B89] Pines A. Aronson E. (1988). Career Burnout: Causes and Cures. New York: Free Press.

[B90] Pretty G. M. H. McCarthy M. E. Catano V. M. (1992). Psychological environments and burnout: gender considerations within the corporation. J. Organ. Behav. 13, 701–711. 10.1002/job.4030130706

[B91] Pyhältö K. Pietarinen J. Haverinen K. Tikkanen L. Soini T. (2021). Teacher burnout profiles and proactive strategies. Eur. J. Psychol. Educ. 36, 219–242. 10.1007/s10212-020-00465-6

[B92] Ramos D. K. Anastácio B. S. da Silva G. A. Rosso L. U. Mattar J. (2023). Burnout syndrome in different teaching levels during the COVID-19 pandemic in Brazil. BMC Public Health 23:235. 10.1186/s12889-023-15134-8 36737710 PMC9896436

[B93] Ronfeldt M. Farmer S. McQueen K. Grissom J. (2015). Teacher collaboration in instructional teams and student achievement. Am. Educ. Res. J. 52, 475–514. 10.3102/000283121558556238293548

[B94] Sadoughi M. Hejazi S. Y. Khajavy G. H. (2024). Protecting language teachers from burnout: the roles of teaching mindset, teaching grit, and emotion regulation. Lang. Teach. Res. 1–23. 10.1177/13621688241238350

[B95] Sak R. (2018). Gender differences in Turkish early childhood teachers' job satisfaction, job burnout and organizational cynicism. Early Child. Educ. J. 46, 643–653. 10.1007/s10643-018-0895-9

[B96] Sánchez-Pujalte L. Mateu D. N. Etchezahar E. Gómez Yepes T. (2021). Teachers' burnout during COVID-19 pandemic in Spain: trait emotional intelligence and socioemotional competencies. Sustainability 13:7259. 10.3390/su13137259

[B97] Schaufeli W. Enzmann D. (1998). The Burnout Companion to Study and Practice: A Critical Analysis. London: Taylor and Francis.

[B98] Schaufeli W. B. Bakker A. B. (2004). Job demands, job resources, and their relationship with burnout and engagement: a multi-sample study. J. Organ. Behav. 25, 293–315. 10.1002/job.248

[B99] Scheuch K. Haufe E. Seibt R. (2015). Teachers' health. Dtsch. Arztebl. Int. 112, 347–356. 10.3238/arztebl.2015.0347 26051692 PMC4558646

[B100] Schwab R. L. Iwanicki E. F. (1982). Perceived role conflict, role ambiguity, and teacher burnout. Educ. Adminis. Q. 18, 60–74. 10.1177/0013161X82018001005

[B101] Shen B. McCaughtry N. Martin J. Garn A. Kulik N. Fahlman M.. (2015). The relationship between teacher burnout and student motivation. Br. J. Educ. Psychol. 85, 519–532. 10.1111/bjep.12089 26235820

[B102] Shirom A. (1989). “Burnout in work organizations,” in International Review of Industrial and Organizational Psychology, eds. Cooper C. L. Robertson I. T. (New York: John Wiley and Sons).

[B103] Skaalvik E. M. Skaalvik S. (2007). Dimensions of teacher self-efficacy and relations with strain factors, perceived collective teacher efficacy, and teacher burnout. J. Educ. Psychol. 99, 611–625. 10.1037/0022-0663.99.3.611

[B104] Skaalvik E. M. Skaalvik S. (2009). Does school context matter? Relations with teacher burnout and job satisfaction. Teach. Teacher Educ. 25, 518–524. 10.1016/j.tate.2008.12.006

[B105] Skaalvik E. M. Skaalvik S. (2010). Teacher self-efficacy and teacher burnout: a study of relations. Teach. Teacher Educ. 26, 1059–1069. 10.1016/j.tate.2009.11.001

[B106] Skaalvik E. M. Skaalvik S. (2011). Teacher job satisfaction and motivation to leave the teaching profession: relations with context, feeling of belonging and emotional exhaustion. Teach. Teach. Educ. 27, 1029–1038. 10.1016/j.tate.2011.04.001

[B107] Smylie M. A. (1999). “Teacher stress in a time of reform”, in Understanding and Preventing Teacher Burnout: A Sourcebook of International Research and Practice, eds. Vandenderghe R. Huberman A. M. (New York: Cambridge University Press).

[B108] Steinhardt M. A. Jaggars S. E. S. Faulk K. E. Gloria C. T. (2011). Chronic work stress and depressive symptoms: assessing the mediating role of teacher burnout. Stress Health 27, 420–429. 10.1002/smi.1394

[B109] Stengård J. Mellner C. Toivanen S. Nyberg A. (2022). Gender differences in the work and home spheres for teachers, and longitudinal associations with depressive symptoms in a Swedish cohort. Sex Roles 86, 159–178. 10.1007/s11199-021-01261-2

[B110] Sterrett W. Sclater K. Murray B. (2011). Preemptive relationships: teacher leadership in strengthening a school community. Virgin. Educ. Leadersh. 8, 17–26.

[B111] Sun W. Dapat R. (2024). Unraveling job stress, burnout, and psychological capital among Chinese EFL teachers in higher institutions. Int. Educ. Stud. 17, 29–44. 10.5539/ies.v17n5p29

[B112] Sutton R. E. Wheatley K. F. (2003). Teachers' emotions and teaching: a review of the literature and directions for future research. Educ. Psychol. Rev. 15, 327–358. 10.1023/A:1026131715856

[B113] Tang Y. He W. (2022). Emotion regulation and psychological capital of Chinese University Students during the COVID-19 pandemic: the serial mediation effect of learning satisfaction and learning engagement. Int. J. Environ. Res. Public Health 19:13661. 10.3390/ijerph192013661 36294240 PMC9603398

[B114] Taxer J. L. Gross J. J. (2018). Emotion regulation in teachers: the “why” and “how.” Teach. Teach. Educ. 74, 180–189. 10.1016/j.tate.2018.05.008

[B115] Tsouloupas C. N. Carson R. L. Matthews R. Grawitch M. J. Barber L. K. (2010). Exploring the association between teachers' perceived student misbehaviour and emotional exhaustion: the importance of teacher efficacy beliefs and emotion regulation. Educ. Psychol. 30, 173–189. 10.1080/01443410903494460

[B116] Wang X. Motevalli S. Huang W. (2025). How gamification affordances reduce academic burnout in online learning: the mediating role of positive psychological capital. Interact. Technol. Smart Educ. 10.1108/ITSE-09-2024-0222

[B117] Wang Y. Guan H. (2020). Exploring demotivation factors of Chinese learners of English as a foreign language based on positive psychology. Revista Argentina de Clinica Psicologica, 29, 851–861. 10.24205/03276716.2020.116

[B118] Xu L. (2017). Teacher-researcher role conflict and burnout among Chinese university teachers: a job demand-resources model perspective. Stud. High. Educ. 44, 903–19. 10.1080/03075079.2017.1399261

[B119] Yang D. (2022). Unpacking the role of Chinese EFL teacher aggression and burnout in their professional success: a teachers' psychology perspective. Front. Psychol. 13:1001252. 10.3389/fpsyg.2022.1001252 36186363 PMC9521366

[B120] Yang X. Du J. (2024). The effect of teacher self-efficacy, online pedagogical and content knowledge, and emotion regulation on teacher digital burnout: a mediation model. BMC Psychol. 12, 1–14. 10.1186/s40359-024-01540-z 38279159 PMC10811882

[B121] Zeichner K. M. Liston D. P. (2013). Reflective Teaching: An Introduction. New York: Routeledge.

[B122] Zhang L. J. Fathi J. Mohammaddokht F. (2023). Predicting teaching enjoyment from teachers' perceived school climate, self-efficacy, and psychological wellbeing at work: EFL teachers. Percept. Mot. Skills 130, 2269–2299. 10.1177/00315125231182269 37395156 PMC10552353

[B123] Zhang Y. Zhang S. Hua W. (2019). The impact of psychological capital and occupational stress on teacher burnout: mediating role of coping styles. Asia Pac. Educ. Res. 28, 339–349. 10.1007/s40299-019-00446-4

